# Effect of Two Different Stunning Methods on the Quality Traits of Rabbit Meat

**DOI:** 10.3390/ani10040700

**Published:** 2020-04-17

**Authors:** Joanna Składanowska-Baryza, Agnieszka Ludwiczak, Ewa Pruszyńska-Oszmałek, Paweł Kołodziejski, Marek Stanisz

**Affiliations:** 1 Department of Animal Breeding and Product Quality Assessment, Faculty of Veterinary Medicine and Animal Science, Poznań University of Life Sciences, Słoneczna 1, 62-002 Suchy Las, Poland; agnieszka.ludwiczak@up.poznan.pl (A.L.); stanisz@up.poznan.pl (M.S.); 2 Department of Animal Physiology and Biochemistry, Faculty of Veterinary Medicine and Animal Science, Poznań University of Life Sciences, 35 Wołyńska Street, 60-637 Poznań, Poland; ewaprusz@up.poznan.pl (E.P.-O.); pawel.kolodziejski@up.poznan.pl (P.K.)

**Keywords:** meat, stunning, genotype, rabbit

## Abstract

**Simple Summary:**

According to consumer opinion, poor welfare leads to lower quality animal products. The stunning method plays a very important role as far as animal welfare, meat quality, and public health are concerned. The aim of the presented study was to assess the effect of two different stunning methods on the blood stress indicators and the quality of rabbit meat. The underlying goal was to improve the welfare of rabbits at the time of slaughter. The study was conducted on a commercial rabbit farm in Poland. The hybrids used in this study were obtained crossing Hyplus PS19 females with Hycole males (genotype I) and Jordan × Hycole females with Hyla males (genotype II). According to the presented findings, the genotype groups of rabbits differed in meat performance, but the differences in meat quality were minor. The examination of the blood stress biomarkers showed that both stunning methods are optimal when taking the welfare of rabbits into consideration. The presented research revealed some differences in the physiochemical traits of meat, with only slight differences between genotypes and stunning methods.

**Abstract:**

The aim of the study was to assess the effect of two different stunning methods on the level of blood stress indicators (cortisol, insulin, glucose) and rabbit meat quality. The experiment was conducted on crossbreds of Hycole, Hyla, and Jordan rabbit strains: from mating Hyplus PS19 females with Hycole males (genotype I, n = 20) and Jordan × Hycole females with Hyla males (genotype II, n = 20). Prior to slaughter, the animals were weighed and divided into two groups according to the stunning method: 10 rabbits from each genetic group were stunned with an electric stunner, and the remaining 10 were stunned mechanically using a club. Genotype II was characterised by higher body weight at slaughter (*p* < 0.05), hot dressing percentage (*p* < 0.05), cold dressing percentage (*p* < 0.05), hot carcass weight (*p* < 0.05), and cold carcass weight (*p* < 0.05), compared to genotype I. The stunning method slightly influenced the meat lightness (*p* = 0.035). The meat of electrically stunned rabbits was characterised with higher drip loss (*p* < 0.0001) and lower plasticity (*p* = 0.043). Among the analysed traits of meat, only the drip loss (*p* = 0.014) and the percentage of extractable fat were affected by genotype (*p* = 0.044). Neither the stunning method nor the genotype affected rabbit meat texture characteristics. The study was undertaken because of the increasing importance of rabbit meat production as a developing sector of the meat industry and the need to improve the welfare of rabbits by selecting the most acceptable slaughter methods for these animals. To conclude, the analysed rabbit meat was characterised with good quality. There were only slight differences between genotypes and the stunning groups.

## 1. Introduction

The quality of rabbit meat is affected by many factors, such as breed [1], feeding [2], and transport [3,4], as well as the stunning and slaughter method [5]. Nowadays, the consumer focus is not only on the dietary value of meat but also on the way the meat is obtained [2]. The production of rabbits, especially in Western Europe, is subject to increasing consumer criticism. The main focus has been on farm conditions as well as the ante-mortem phase of the production chain [6,7]. The stunning method plays a very important role throughout the whole production chain from animal welfare to meat quality and public health [7]. According to consumer opinion, poor welfare leads to a lower animal product quality. Today, how farm animals are cared for affects the decision of whether or not to purchase the final product [8,9]. The most popular slaughter methods used in European slaughterhouses are industrial slaughter (stunning must precede the bleeding of the animal) and halal and kosher slaughter (without stunning). Stunning the animal before slaughter is a mandatory procedure in the European Union, USA, Australia, New Zealand, South Africa, Brazil, and East Asia Countries, but it is not permissible in the majority of Muslim countries. Although there are exceptions, a number of stunning methods have been accepted by Malaysia, Indonesia, and some Muslim communities in the UK, Sweden, Germany, Norway, Switzerland, and Denmark, as long as the animal is stunned but alive until it is butchered [10]. The slaughter procedures performed without stunning are controversial in terms of animal welfare. Stunning is not as controversial because it ensures insensibility to pain until exsanguination [10]. In 2009, the European Union (EU) Slaughter Regulation permitted religious slaughter without stunning. There is no wide regulation in the EU on slaughter without stunning; individual countries decide for themselves. The total ban on ritual slaughter applies only in Sweden, while in Poland, Denmark, and Greece, it can be done after an animal has been previously stunned. A similar solution is being planned by Slovenia. In other EU countries, certain restrictions apply, for example; that an animal may be stunned or anaesthetised during or shortly after slaughter, or an official exemption from the ban on stunning or anaesthetising may be granted for the Muslim and Jewish communities [10,11,12] Stunning is achieved by using a mechanical or electrical device or by using a gas, such as carbon dioxide (narcosis) [6]. In electrical stunning, the most frequently used voltage and power are 49 V and 250 Hz. Electrically stunned rabbits receive an electroshock lasting approximately 2 sec, in the frontal sinus (*fossa temporalis*) with a “V”-shaped metal electrode [13]. A mechanical stun involves a rapid disruption of brain function by a captive bold (penetrative or non-penetrative) narrow rod or hammer. Adequate training in slaughterhouse practices should be required to make sure that the slaughter procedures are carried out by people with an appropriate level of competence [14]. Stress is an important factor affecting meat quality. It is important that both the genotype of animals, and the environment (transport, stunning method, pre-slaughter handling) promote the lowest possible level of stress. The rabbit industry offers a wide variety of synthetic lines characterised by very good production traits. The genetics of synthetic rabbit strains change dynamically. One can observe the improvement of the leading strains and discontinuance in the production of less popular ones [15,16,17,18]. Available rabbit genotypes may differ in their level of susceptibility to stress [1,3]. The aim of this study was to assess the effect of two different stunning methods on the blood stress indicators and the quality of rabbit meat. The underlying goal of the research was to improve rabbit welfare at the time of slaughter. The study was conducted on a commercial rabbit farm. In this study, the breeders used for the production of fryers were not pure rabbit strains. They were crossbreds of the leading synthetic rabbit lines, characterised with high maternal qualities in the earlier production cycles on this farm.

## 2. Materials and Methods

### 2.1. Animals

The study was conducted on 40 hybrid rabbits (males) from mating Hyplus PS19 females with Hycole males (genotype I) and Jordan × Hycole females with Hyla males (genotype II) (Table 1). Up to the slaughter age, the animals were raised in the same rabbitry and were fed the same diet. The rabbits were fattened in a conventional system based on collective full-wire cages, six rabbits per cage. A single cage was 40 cm × 85 cm × 35 cm (width × length × height). The fattened rabbits were kept under the lighting schedule of 12/12 h, at the light intensity of 50 lux, and the temperature 16–18 °C. During the fattening period, the rabbits were fed granulated commercial feed containing 16.0% of total protein, 14.0% of crude fibre, and 10.4 MJ of metabolic energy. The rabbits had unlimited access to water and hay. All rabbits in the experiment were slaughtered at the age of 86 days. Both genotype groups were fasted overnight with unlimited access to water and slaughtered the next morning. Prior to slaughter, the animals were weighed and divided into two groups according to the stunning method: 10 rabbits from each genetic group were stunned with electric stunner using 49 V current for 15 sec, and remaining 10 were stunned mechanically using a club. Stunning was immediately followed by cutting the jugular veins (according to European Union Law Gazette 2009, No. 1099/2009; Regulation of European Union Council [19]). The animals were hanged by the hind limbs in order to allow bleeding out. Twenty-four hours post-mortem, the carcasses were weighed (cold carcass weight, kg), and the right and left *musculus longissimus thoracis et lumborum* (LTL) and *musculus biceps femoris* (BF) were cut from the carcasses in order to examine the quality of rabbit meat. During the analysis, the muscles were kept at +2 °C. The dressing percentage was calculated as a relation of the cold carcass weight to the pre-slaughter body weight.

### 2.2. Blood Stress Biomarkers

The levels of rabbits’ responses to stress caused by different stunning methods were evaluated using the baseline serum levels of cortisol, insulin, glucose, total cholesterol, triglycerides, non-esterified fatty acids (NEFA), and protein. The blood was caught in plastic test tubes from the jugular veins of animals, after they were slaughtered and hanged by their hind limbs to drain. The blood samples were clotting for 30 min and next were centrifuged (Heraeus Megafuge 40R, R-404A, ThermoFisher, Osterode am Harz, Germany) at 14,659× *g* for 15 min at 4 °C. Then, the separated serum was transferred to new tubes and stored at −20 °C until further processing. The metabolic profile was defined using commercially available enzymatic colorimetric diagnostic tests according to the manufacturer’s instructions. Triglycerides (cat. no. T7531), total cholesterol (cat. no. C7509), glucose (cat. no. G7519), and total protein (cat. no. T7528) levels were determined using Pointe Scientific reagent kits (Pointe Scientific Polska Sp. z o.o., Warsaw, Poland). Concentration of NEFA was measured using a NEFA determination kit (HR Series NEFA-HR(2), Richmond, VA, USA) manufactured by WAKO (Wako Diagnostics, Richmond, VA, USA). The optical density of the samples was determined using a Synery 2 (BioTek, Winooski, VT, USA) microplate reader. The serum hormonal profile was determined using radioimmunoassay (RIA) kits. All procedures were performed following the recommendations of the manufacturer. Cortisol was assayed using the Cortisol RIA kit (cat. no. IM1841, Beckman Coulter, Prague, Czech Republic). Insulin was determined using the Insulin RIA kit (cat. No. HI-14K. Merck Millipore, Burlington, VT, USA). The radioactivity of the samples was measured with a Wallac Wizard 1470 Gamma Counter (Perkin Elmer, Waltham, MA, USA).

### 2.3. Quality of Rabbit Meat

#### 2.3.1. Acidity Measures

The pH was measured by inserting a calibrated combined glass-calomel electrode (Lo 406-M6-DXK-S7/25, Mettler Toledo, Columbus, OH, USA) connected to a portable pH meter (type 1140, Mettler Toledo, Columbus, OH, USA,) into the meat. The pH was measured 45 min (pH_45min_), 24 (pH_24h_) and 48 h (pH_48h_) post-mortem in the LTL and BF.

#### 2.3.2. Colour Measurements

The first colour measurements were recorded 45 min post-mortem on the muscle surface. The colour was measured using a portable spectrophotometer CM 700d (Konica Minolta, Amsterdam, The Netherlands) (illuminant D65, 10° observer with a 8 mm diameter aperture size). The tristimulus CIE system which measures lightness (L*), redness (a*), and yellowness (b*) was used. Colour measurements were repeated 24 and 48 h post-mortem, after storage at +2 °C.

#### 2.3.3. Capacity to Hold Residual Water and Water Fractions

The total water, free water, and plasticity were measured on samples of LTL and BF. The drip loss and cooking loss were measured only on the LTL. All the aforementioned measurements were taken 24 h post-mortem. 

The drip loss (%) was measured according to the method of Honikel [20]. The 3 cm thick, transverse slices of the analysed muscles (25–30 g) were weighed, hung on hooks, and placed in a container to reduce evaporation (+2 °C). After 24 h, the samples were reweighed to calculate the change in the weight.

The free water (%) was measured using a filter paper press method, according to the method of Grau and Hamm [21], in modification of Pohja and Niinivaara [22]. Samples (0.280–0.320 g) of ground meat were placed on a filter paper between two glass tiles. A force of 2 kg was applied on each sample for 5 min. Then, the samples were removed from the filter paper and reweighed straight after to calculate the change in the weight. The calculations were made using the following formula:

Free water (%) = (sample of ground meat − sample of meat after 5 min of 2 kg pressure) × 100/sample of ground meat.(1)

Meat plasticity (cm^2^) was expressed as the area of the compressed meat sample used for the measurement of free water, according to the method of Grau and Hamm [21]. The plasticity was expressed as the area of the compressed meat sample used for the measurement of free water.

The cooking loss (%) was measured according to the method of Honikel [23]. The 3 cm thick, transverse slices of the analysed muscles (25–30 g) were placed in thin polyethylene bags, with the bag’s wall firmly adhered to the meat sample. The bags with meat were placed in a water bath at 90 °C (for about 30 min) until reaching the core temperature of 75 °C (measured with the thermocouples). Then, the samples were cooled to room temperature and reweighed after removing the excess of moisture with a paper towel. The change in the weight of the sample was calculated (%).

#### 2.3.4. Texture Analyses

The texture assessment included the measurements of forces and energy used to cut through the sample, and the forces used to bite through the sample. The cutting forces and cutting energy measurements were made with the Warner-Bratzler V-shaped blade attached to the TA.XT Plus Texture Analyser (Stable 136 Micro Systems, Warrington, UK), (test speed 2 mm/s; distance 20 mm; force 20 g). In order to analyse the cutting forces and cutting energy of raw material, the meat after drip loss measurement was used. The other measurements were made on meat samples after the measurement of cooking loss (kept after thermal processing for 24 h). Prior to analysis, the muscle samples were placed at a room temperature, and 3 cores (1.6 cm diameter) were removed from each sample with a round knife, parallel to the muscle fibres. The Warner-Bratzler measurements included the force used at the onset of cutting (WB-O; N), the peak force (WB-P; N), and the area under the curve, which is referred to as the shear energy (WB-E; N/mm). The measurement of biting (the force used to bite through the sample) was done on the cooked samples with Volodkevich Bite Jaws (test speed 2 mm/s; strain 100%; force 5 g). The meat samples were obtained from the muscle parts used for the measurement of cooking loss, which were subjected to chilled storage for 24 h at 2 °C in plastic bags. Prior to analysis, the muscle parts were placed at room temperature. The Twin Blade Sample Preparation Tool was used to cut 10 mm × 10 mm size stripes laterally to the muscle fibers (3 stripes per muscle). The following measurements were taken: the force measured at the onset of biting (V-O; N) and the peak force (V-P; N).

#### 2.3.5. Chemical Composition

The chemical composition of rabbit meat was examined according to AOAC (Association of Official Analytical Chemists) methods [5]. The analyses were made 24 h post-mortem and included the determination of dry matter content (the samples were dried at 105 °C to a constant weight), the determination of the total protein content with the Kjeldahl procedure (K-424 Buchi digestion unit; Büchi Labortechnik AG, Flawil, Switzerland; Schott TitroLine, SCHOTT, Mainz, Germany), and the determination of extractable fat content by Soxhlet extraction with diethyl ether (MLL 147, AJL Electronics, Cracow, Poland). The determination of dry mater content allowed calculating the percentage of total water.

### 2.4. Statistical Analysis

The effect of the genotype on the body weight at slaughter was calculated using the one-way ANOVA of SAS ver.9.4 (SAS Institute Inc., Cary, NC, USA) [24].

The effect of the genotype on the hot carcass dressing percentage, cold carcass dressing percentage, hot and cold carcass weight were calculated with the PROC GLM (general linear models) model given below.

Y_ik_ = µ + δ_i_ + bw_ik_ + e_ik_ (2)

where:

µ—the overall mean of the analysed trait,
δ_i_—the fixed effect of the genotype (i = 1, 2),
b—the partial linear regression coefficient,
w_ik_—the body weight at slaughter of animal,
e_ik_—the random error.

The effect of the stunning method and genotype on the blood stress indicators (level of insulin, cortisol, glucose, cholesterol, triglycerides, NEFA, protein), drip loss, cooking loss, V-O (the force at the onset of biting), and V-P (biting peak force) was calculated with the PROC GLM model given below.

Y_ijk_ = µ + α_j_ + δ_i_ + (αδ)_ji_ + bw_ijk_ + e_ijk_ (3)

where:

µ—the overall mean of the analysed trait,
α_j_—the fixed effect of the stunning method (j = 1, 2),
δ_i_—the fixed effect of the genotype (i = 1, 2),
(αδ)_ji_—interaction (stunning method × genotype),
b—the partial linear regression coefficient,
w_ijk_—the body weight at slaughter,
e_ijk_—the random error.

The effect of the stunning method, genotype, muscle type, and time post-mortem on the pH (pH_45min_, pH_24h_, and pH_48h_) and muscle colour L* (L*4_5min_, L*_24h_, and L*_48h_), a* (a*_45min_, a*_24h_ and a*_48h_) and b* (b*_45min_, b*_24h_ and b*_48h_) was calculated with the PROC MIXED (mixed models) model given below.

y_ijklm_ = µ + δ_i_ + α_j_ + π_k_ + γ_k(l)_ + β_k(l)(m)_ + (δαγβ)_jilm_ + bw_ijkl_ + e_ijklm_ (4)

where:

µ—the overall mean of the analysed trait,
α_j_—the fixed effect of the stunning method (j = 1, 2),
δ_i_—the fixed effect of the genotype (i = 1, 2),
π_k_—the random effect of the animal
γ_k(l)_—the random effects of the muscle (l = 1, 2) nested in the animal,
β_k(l)(m)_—the effect of the time post-mortem (m = 1, 2, 3) as the repeated measures nested in the l^th^ muscle nested in the animal,
(δαγβ)_jilm_—interaction (stunning method × genotype × muscle × time post-mortem),
b—the partial linear regression coefficient,
w_ijkl_—the body weight at slaughter,
e_ijklm_—the random error.

The effect of the stunning method, genotype, and muscle type on the total water, plasticity, dry matter, crude protein, extractable fat, and water/crude protein ratio was calculated with the PROC MIXED (mixed models) model given below.

y_ijkl_ = µ + δ_i_ + α_j_ + π_k_ + γ_k(l)_ + (δαγ)_jil_ + bw_ijkl_ + e_ijkl_ (5)

where:

µ—the overall mean of the analysed trait,
α_j_—the fixed effect of the stunning method (j = 1, 2),
δ_i_—the fixed effect of the genotype (i = 1, 2),
π_k_—the random effect of the animal
γ_k(l)_—the random effects of the muscle nested in the animal,
(δαγ)_jil_—interaction (stunning method × genotype × muscle),
b—the partial linear regression coefficient,
w_ijkl_—the body weight at slaughter,
e_ijkl_—the random error.

The effect of the stunning method, genotype, and thermal processing on the WB-O (Warner-Bratzler force on the onset of cutting), WB-P (Warner-Bratzler peak force), and WB-E (Warner-Bratzler shear energy) was calculated with the PROC GLM model given below.

y_ijkl_ = µ + δ_i_ + α_j_ + ε_k_ + (δαε)_jik_ + bw_ijk_ + e_ijkl_ (6)

where:

µ—the overall mean of the analysed trait,
α_j_—the fixed effect of the stunning method (j = 1, 2),
δ_i_—the fixed effect of the genotype (i = 1, 2),
ε_k_—the fixed effect of the thermal processing (k = 1, 2),
(δαε)_jik_–interaction (stunning method × genotype × thermal processing),
b—the partial linear regression coefficient,
w_ijk_—the body weight at slaughter,
e_ijkl_—the random error.

There were no significant interactions between the analysed effects; therefore, the *p*-values of these interactions were not given in the tables. All of the statistical analyses were made with SAS (SAS Institute Inc., Cary, NC, USA) [24]. An analysis of covariance with the body weight at slaughter as covariant was implemented. Tukey–Kramer adjustment was implemented for multiple comparisons of LS (Least squares means) mean differences.

## 3. Results

### 3.1. Meat Performance of the Analysed Rabbit Crossbreds

As expected, the genotype group significantly differed in body weight at slaughter (*p* = 0.003), hot dressing percentage (*p* = 0.029), cold dressing percentage (*p* = 0.033), hot carcass weight (*p* = 0.042), and cold carcass weight (*p* = 0.048) (Table 2). All the meat performance characteristics were better developed in genotype II compared to genotype I.

### 3.2. Stress Biomarkers—Metabolic Blood Parameters and Hormones

In the presented study, the stunning method did not affect the level of stress hormones (Table 3). The genotype groups significantly differed in the level of blood glucose and NEFA, but the concentration of insulin, cortisol, total cholesterol, triglyceride, and protein in the blood of both genotype groups was similar. The rabbits from genotype I were characterised by a higher level of blood glucose (*p* = 0.047) and lower concentration of NEFA (*p* = 0.016), compared to rabbits from genotype II. These results underline the importance of selecting animals, which have a high resistance to stress, as the genotype decides about the level of reaction to stressors such as transport (unavoidable in rabbit production) and slaughter procedures. The body weight of the analysed rabbits affected only the NEFA level (*p* = 0.010) in the blood serum.

### 3.3. Meat pH

According to the results, the pH of rabbit meat was not influenced by the genotype (*p* = 0.797), stunning method (*p* = 0.735), or muscle type (*p* = 0.183) (Table 4). We did not find differences in the meat pH value between genotype groups. In the presented study, the time of the post-mortem affected the pH value of rabbit meat (*p* < 0.001) (Table 4). This observation is an obvious result of the post-mortem muscle biochemistry.

### 3.4. Meat Colour

In our study, the meat brightness was affected by the stunning method (*p* = 0.035) and the time of the post-mortem (*p* = 0.049), with no differences between the genotypes (*p* = 0.970) and muscles (*p* = 0.504) (Table 4). The meat redness (a*) was not affected by the stunning method (*p* = 0.527), the genotype (*p* = 0.539), the time of the post-mortem (*p* = 0.312), and the muscle (*p* = 0.492). The b* index was influenced only by the time of the post-mortem (*p* = 0.014) and ranged from 4.02 to 7.42 units (Table 4).

### 3.5. Water Fraction and the Capacity to Hold Residual Water

The drip loss in the presented study was influenced by the genotype, and it was higher in meat from genotype group II compared to group I (*p* = 0.014). Moreover, the meat from electrically stunned rabbits was characterised with higher drip loss (*p* < 0.0001) and lower plasticity (*p* = 0.043) compared to the meat from the mechanically stunned animals (Table 5). The cooking-loss measures of rabbit meat were not affected by the genotype (*p* = 0.061) or by the stunning method (*p* = 0.366). The major factor determining the rabbit meat capacity to hold residual water and the share of water fractions was the muscle type. The total water and the percentage of free water were higher in BF compared to LTL (*p* < 0.0001, *p* = 0.004 and *p* = 0.007). The presented results clearly show the negative effect of mechanical stunning on drip loss in rabbit meat.

### 3.6. Texture Measurement

The texture measurements are presented in Table 6. According to the results of our research, the WB-O, WB-P, and WB-E values were not affected by the genotype or the stunning method, but only by thermal processing (raw versus cooked meat; *p* < 0.0001). In the presented study, no differences in biting between genotypes, stunning methods, and body weights (*p* > 0.05) were found.

### 3.7. Chemical Composition

The chemical composition of rabbit meat was not affected by different stunning methods. The genotype groups analysed in our study had different percentages of extractable fat in meat (*p* = 0.044). In accordance with Table 7, the major factor determining the chemical composition of rabbit meat analysed in the present research was the muscle type Table 7. The dry matter and crude protein content was higher in LTL compared to BF (*p* < 0.0001). The percentage of extractable fat and the water/crude protein ratio was higher in BF compared to LTL (*p* < 0.0001). These results are due to differences in the microstructure of the analysed muscles.

## 4. Discussion

Since every farm has different environmental conditions, it is difficult to compare the various research results of meat performance analysis [25,26,27]. Similarly to our results, Metzger et al. [26], Tumova et al. [28], and Szendro et al. [29] indicated an effect of genotype on body weight at slaughter, hot carcass weight, and the dressing out percentage of hybrid rabbits. Szendro et al. [29] found that large-sized lines selected for high growth had poorer dressing out percentages than the smaller-sized lines. According to the research results of Apata et al. [6], gas stunning accelerates high blood drain from rabbit carcasses; therefore, the dressed weight, dressing percentage, chilled carcass weight, and chilling loss were higher in the group of rabbits electrically stunned compared to those subjected to gas stunning (*p* > 0.05). In our study, the effect of the stunning method on the slaughter traits of rabbits was not analysed. It is very important to minimise stress by maintaining an optimal level of welfare. Moreover, rabbits should be selected for high adaptability to the possible stressors in the production cycle [18]. Our results and other research results indicate that the stunning method may affect the blood stress biomarkers; however, the magnitude of these changes differs. Nakyinsige et al. [30] observed that the level of blood glucose was significantly higher in the group stunned with gas compared to the halal slaughtered group (*p* < 0.05). Furthermore, they found that it is the carbon dioxide gas used during the stun that increases the anaerobic oxidative metabolism, which in turn increases the level of glucose in the blood. The researchers [30] found that both methods (halal slaughter and gas stunning) caused hypercalcaemia, hyperglycaemia, and increased activities of enzymes, including LDH (lactate dehydrogenase), creatine kinase and alanine transaminase, lactic acidaemia, and leucocytosis. The stress indicator levels of Czech local breeds and commercial hybrids were compared by Tumova et al. [28]. Their results were the opposite of ours; they found that only cholesterol concentration was significantly affected by the genotype (*p* = 0.041), with the highest values observed in Hyplus rabbits (4.2 mmol/L). Four pure rabbit breeds and their crosses raised under Egyptian environmental conditions were compared by Abdel-Azeem et al. [1]. They observed a highly significant effect of genotype on the concentration of protein and triglycerides. The genotypes of rabbits used in our study also differed in their reaction to stress; however, the stressor was not the temperature but the stunning method used. This observation proves that the process of properly directed selection may improve the welfare of rabbits by decreasing their susceptibility to stress. In good quality rabbit meat, the pH_u_ (pH ultimate) value measured after 24 h from the slaughter is above 5.8. Abnormal direction and rapid pH changes are followed by unwanted changes in colour and water-holding capacity [3]. Compared to our study, Nakyinsige et al. [30] observed similar pH changes in the meat of gas-stunned and halal-slaughtered rabbits. Although we did not find any differences in the meat pH value between genotype groups, Barron et al. [15] and Ouhayoun [31] reported that the breed may influence the pH value of rabbit meat. As with our results, Gasperlin et al. and Metzger et al. [26,32] have not found an effect of genotype on the pH of rabbit meat. No effect of the genotype and the stunning method on the pH was found in our study. Nonetheless, the rabbits used for meat production should be selected for high adaptability to the environment to prevent the possible negative effect of different stressors on meat quality. Meat colour depends on different factors: pH decrease, oxygen consumption, metmyoglobin reduction, and the ante mortem stress causing changes in the course of the biochemical reactions in the post-mortem muscle [33,34]. As with our results, Nakyinsige et al. [30] also found different L* values of meat from rabbits stunned using different methods. They observed significantly greater meat lightness in the rabbit group stunned with gas (47.5) compared to the halal slaughtered group (45.6). On the other hand, Dal Bosco et al. [14] claimed that the influence of the stunning method on colour parameter was not relevant (*p* > 0.05). As in our study, Nakyinsige et al. [30] reported that the a* and b* parameters of rabbit meat did not differ between the groups of rabbits stunned using different methods. There are discrepancies between the results of different studies, but the effect of the stunning method on the brightness of rabbit meat found in our presented study is an important observation and may affect the consumer acceptance of this raw animal product. Dal Bosco et al. [14] and Van der Wal et al. [11] concluded that the rabbit meat production chain is very complex, with numerous factors affecting the water factions and capacity of muscle tissue to hold residual water. Such an observation makes it difficult to determine the true effect of the stunning technique on the mentioned traits. It is difficult to propose a standard procedure for measuring the capacity to hold water and for measuring the water fractions in meat. There are too many variations present in the published studies, so it is difficult to compare results [25]. In their study, Apata et al. [6] reported lower values of cooking loss and drip loss in the meat of rabbits stunned with gas compared to animals stunned electrically (*p* < 0.05). Meanwhile, Nakyinsige et al. [30] found that the meat of rabbits submitted to the halal slaughter was characterised with significantly lower cooking loss compared to the gas-stunned rabbits (23.27% versus 25.70% for 24 h and 20.43% versus 24.51% for 7 days). Opposite to our results, Belichovska et al. [25] found that losses during rabbit meat boiling and roasting were lower in the crossbreds than in pure breeds (*p* < 0.05). The above findings are in disagreement with studies conducted by Gil et al. [35], who reported that the textural properties of rabbit loin were affected by genotype. Pascual and Pla [36] found that the value of the cutting force for rabbit meat is 36 N/cm^2^, with no influence of genotype. In our study, no differences in biting between genotypes and stunning methods (*p* > 0.05) were found (Table 6). Unlike our results, Apata et al. [6] found that the stunning method may influence the content of some chemical components of rabbit meat. The effect of genotype on the proximal chemical composition of rabbit meat is more likely, and it has been analysed by many authors. In their research, Metzger et al. [26] compared the meat quality of Hyplus hybrids, purebred Pannon White, and their crossbreds. They reported that meat samples from later maturing rabbits and those of greater body size tend to contain less extractable fat compared to smaller and early maturing animals. Gasperlin et al. [32] found that the genotype has a significant impact on the protein (*p* < 0.05), extractable fat (*p* < 0.05), and moisture (*p* < 0.01) content in rabbit meat. Tumova et al. [28], and Belichovska et al. [25] found that the genotype did not affect the chemical composition of meat from different synthetic lines of rabbits, which is the opposite of our results and the results of other authors.

## 5. Conclusions

According to our presented research results, the genotype groups of rabbits differed in meat performance. As far as the blood stress biomarkers are concerned, both methods proved optimal when considering the welfare of rabbits. The presented research revealed some differences in the physiochemical traits of meat, and these differences were related to the stunning method. The meat from mechanically stunned animals had significantly higher drip loss and plasticity compared to those electrically stunned. Moreover, the meat from the different stunning groups differed in colour brightness. Therefore, from the perspective of the rabbit meat colour and ability to hold residual water (traits that affect the consumer acceptance of meat), electrical stunning is superior to mechanical stunning of rabbits.

## Figures and Tables

**Table 1:** The number of animals according to the stunning method and genotype.

Mechanical (n = 20)		Electrical (n = 20)	
Genotype I (G I) n = 10	Genotype II (G II) n = 10	Genotype I (G I) n = 10	Genotype II (G II) n = 10
Hyplus PS19 × Hycole	(Jordan × Hycole) × Hyla	Hyplus PS19 × Hycole	(Jordan × Hycole) × Hyla

n—number of animals; PS19—Hyplus parental female line.

**Table 2:** The effect of genotype and body weight on rabbit meat performance (SEM: standard error of the mean).

Trait	Genotype			Effect (*p*-Value)	
	I	II	SEM	Genotype	Body Weight
Body weight at slaughter (kg)	2.65	2.91	0.06	0.003	
Hot carcass dressing percentage (%)	48.53	50.43	0.61	0.029	<0.0001
Cold carcass dressing percentage (%)	46.28	48.08	0.59	0.033	<0.0001
Hot carcass weight (kg)	1.35	1.40	0.02	0.042	0.456
Cold carcass weight (kg)	1.29	1.34	0.02	0.048	0.412

**Table 3:** The effect of stunning methods, genotype, and body weight on the blood levels of stress biomarkers (SEM: standard error of the mean).

Stress Biomarker	Mechanical		Electrical			Effect (*p*-Value)		
	G I	G II	G I	G II	SEM	Stunning Methods	Genotype	Body Weight
Insulin (µU/mL)	18.03	20.17	28.29	19.97	4.91	0.914	0.165	0.563
Cortisol (nmol/L)	20.71	17.15	19.48	20.55	2.25	0.642	0.626	0.430
Glucose (mg/dL)	131.9	123.7	138.6	129.2	5.8	0.131	0.047	0.232
Cholesterol (mg/dL)	88.18	84.19	83.41	95.75	4.36	0.448	0.393	0.648
Triglycerides (mg/dL)	75.27	81.49	77.39	81.34	3.65	0.787	0.209	0.992
NEFA (mmol/L)	0.40	0.54	0.43	0.51	0.04	0.881	0.016	0.010
Protein (g/dL)	6.67	6.64	6.74	6.80	0.09	0.198	0.928	0.753

G I—genotype I; G II—genotype II; NEFA: non-esterified fatty acids.

**Table 4:** The effect of stunning methods, genotype, time post-mortem, muscle, and body weight on the pH and colour measures of rabbit meat (SEM: standard error of the mean).

Trait	Time Post-Mortem		Mechanical				Electrical					Effect (*p*-Value)				
			G I		G II		G I		G II		SEM	Stunning Methods	Genotype	Time Post-Mortem	Muscle	Body Weight
			LTL	BF	LTL	BF	LTL	BF	LTL	BF						
pH	45 min	I	6.67	6.65	6.64	6.63	6.85	6.72	6.86	6.79	0.05	0.735	0.797	0.001	0.183	0.159
	24 h	II	5.75	5.81	5.71	5.88	5.73	5.86	5.74	5.82	0.05					
	48 h	III	5.78	5.82	5.70	5.81	5.71	5.84	5.76	5.81	0.05					
L*	45 min	I	56.5	57.2	54.1	55.2	56.3	56.9	53.8	54.9	0.7	0.035	0.970	0.049	0.504	0.405
	24 h	II	58.1	58.5	55.5	57.1	57.3	58.7	56.3	58.2	0.7					
	48 h	III	59.9	58.9	58.8	57.8	59.9	60.2	56.9	59.1	0.7					
a*	45 min	I	1.38	1.13	1.69	1.32	0.59	0.78	1.65	1.14	0.31	0.527	0.539	0.312	0.492	0.332
	24 h	II	0.81	1.36	0.12	0.76	0.79	0.91	0.56	0.82	0.31					
	48 h	III	0.74	0.84	0.98	0.85	0.65	0.96	1.05	1.15	0.31					
b*	45 min	I	6.87	5.01	5.71	7.42	6.71	5.17	7.31	4.74	0.73	0.196	0.399	0.014	0.831	0.189
	24 h	II	5.84	6.73	4.69	7.21	6.16	5.58	7.08	5.72	0.73					
	48 h	III	5.35	5.28	5.75	6.24	4.02	5.46	4.06	5.50	0.73					

G I—genotype I; G II—genotype II; LTL—*longissimus thoracis et lumborum*, BF—*biceps femoris*; L*—lightness; a*—redness; b*—yellowness.

**Table 5:** The effect of stunning methods, genotype, muscle, and body weight on the water fractions and capacity to hold residual water by rabbit meat (SEM: standard error of the mean).

Trait	Mechanical				Electrical					Effect (*p*-Value)			
	G I		G II		G I		G II		SEM	Stunning Methods	Genotype	Muscle	Body Weight
	LTL	BF	LTL	BF	LTL	BF	LTL	BF					
Drip loss (%)	1.77		1.95		2.15		2.32		0.07	<0.0001	0.014		0.256
Total water (%)	75.9	76.4	75.9	76.5	75.9	76.4	75.9	76.5	0.2	0.986	0.791	<0.0001	0.186
Free water (%)	28.6	31.1	29.2	31.6	29.9	32.4	30.3	32.3	1.2	0.199	0.724	0.004	0.889
Free water share in total water (%)	37.7	40.7	38.4	41.4	39.5	42.4	39.8	42.2	1.5	0.188	0.734	0.007	0.916
Cooking loss (%)	22.1		24.2		22.3		22.9		0.6	0.366	0.061		0.348
Plasticity (cm^2^)	4.03	4.43	4.28	4.35	3.74	3.65	3.78	3.70	0.3	0.043	0.816	0.652	0.695

G I—genotype I; G II—genotype II; LTL—longissimus thoracis et lumborum, BF—biceps femoris.

**Table 6:** The effect of stunning methods, genotype, thermal processing, and body weight on the textural traits of rabbit longissimus thoracis et lumborum (SEM: standard error of the mean).

Trait	Mechanical				Electrical					Effect (*p*-Value)			
	G I		G II		G I		G II		SEM	Stunning Methods	Genotype	Thermal Processing	Body Weight
	Raw	Cooked	Raw	Cooked	Raw	Cooked	Raw	Cooked					
WB-O (N)	6.8	13.5	8.0	13.9	7.7	12.2	7.5	16.5	1.1	0.638	0.103	<0.0001	0.126
WB-P (N)	8.5	15.8	9.5	15.9	10.3	13.6	9.2	16.5	0.9	0.947	0.318	<0.0001	0.206
WB-E (N*mm)	3024	4891	3413	4940	3866	4813	3351	5489	282	0.163	0.517	<0.0001	0.183
V-O (N)		21.4		19.7		21.3		24.1	1.8	0.332	0.810		0.679
V-P (N)		21.6		19.9		21.5		24.4	1.9	0.324	0.795		0.656

G I—genotype I; G II—genotype II; WB-O—Warner-Bratzler force on the onset of cutting; WB-P—Warner-Bratzler peak force; WB-E—Warner-Bratzler shear energy; V-O—the force on the onset of biting; V-P—the biting peak force.

**Table 7:** The effect of stunning methods, genotype, muscle, and body weight on the chemical composition of rabbit meat (SEM: standard error of the mean).

Trait	Mechanical				Electrical					Effect (*p*-Value)			
	G I		G II		G I		G II		SEM	Stunning Methods	Genotype	Muscle	Body Weight
	LTL	BF	LTL	BF	LTL	BF	LTL	BF					
DM (%)	24.10	23.56	24.08	23.53	24.13	23.55	24.06	23.53	0.18	0.988	0.824	<0.0001	0.128
CP (%)	22.48	21.59	22.29	21.86	22.44	21.58	22.30	21.86	0.19	0.918	0.678	<0.0001	0.104
EF (%)	0.56	0.99	0.72	1.15	0.56	1.00	0.72	1.16	0.09	0.999	0.044	<0.0001	0.445
W/CP	3.38	3.54	3.41	3.50	3.38	3.54	3.41	3.50	0.04	0.987	0.735		0.269

DM—dry matter, CP—crude protein, EF—extractable fat, W/CP—water/crude protein ratio; G I—genotype I; G II—genotype II; LTL—*longissimus thoracis et lumborum*, BF—*biceps femoris.*
